# Draft Genome Sequence of *Bacillus subtilis* Strain D7XPN1, Isolated from Commercial Bioreactor-Degrading Food Waste

**DOI:** 10.1128/genomeA.00989-14

**Published:** 2014-10-02

**Authors:** Joseph Adelskov, Bharat K. C. Patel

**Affiliations:** Microbial Gene Research and Resources Facility, School of Natural Sciences, Griffith University, Brisbane, Queensland, Australia

## Abstract

The analysis of the 4.1-Mb draft genome sequence of a moderately thermophilic, heterotrophic, and facultatively anaerobic bacterium, *Bacillus subtilis* strain D7XPN1, identified genes for a range of enzymes with potential in the biodegradation of food waste, a property consistent with the ecological habitat of the isolate.

## GENOME ANNOUNCEMENT

*Bacillus subtilis* strain D7XPN1 (KCTC 33554; JCM 30051) is a heterotrophic, moderately thermophilic (optimal temperature 45°C, temperature growth range between 24 and 50°C), facultative anaerobic, and fermentative bacterium that was isolated on 0.1% tryptic soy broth (TSB) from a sample collected from a food waste–degrading commercial bioreactor called Baku Baku. The sample was collected on day 7 when the *in situ* temperature of the bioreactor was 44°C (1). Strain D7XPN1 was cultured in 0.1% TSB under optimal growth conditions (pH 7.0 and 45°C), the cells were centrifuged, and the DNA from the cell pellet was purified using a modification of Marmur’s method (2). The genomic DNA of strain D7XPN1 that was sequenced by an Ion Torrent PGM sequencer and a 318 chip at the Australian Genome Research Facility (AGRF) core facility generated 722, 222 reads totaling 141 Mbp of sequence data. Genome assembly using GS De Novo Assembler (version 2.9) generated 28 contigs (40× coverage).

The assembled data of 4.1 Mbp, with an average G+C content of 43.8 mol%, was analyzed using the online annotation server and RAST (3), and automatic gene annotation was carried out by the NCBI Prokaryotic Genomes Automatic Annotation Pipeline (PGAAP) (http://www.ncbi.nlm.nih.gov/genome/annotation_prok). The genome sequence comprised 4,021 genes, including 3,033 putative protein-encoding genes, 66 tRNA genes, and 4 rRNA genes (5S rRNA, 16S rRNA, 23S rRNA). PhyloSift version 1.0.0_02 (4) indicated that 66% of the phylogenetic marker genes were related to the genus *Bacillus*, family *Bacillaceaea*, phylum *Firmicutes*, and, in particular, to the *Bacillus subtilis* group (28%) comprising *B. amyloliquefaciens*, *B. vallismortis*, and *B. subtilis*, as well as to the sole member, *Bacillus* species JS (32%). Further BLAST analysis using ANIb, part of the JSpecies suite of programs (5), indicated that strain D7XPN1 was most closely related to *Bacillus* species strain JS (6) with an average nucleotide identify of 98.6%.

A large number of genes involved in carbohydrate degradation (516), including genes for α-amylase, pullulanase, glucosidase, galactosidase, glucuroxylanase, and arabinogalactan endo-1,4-beta-galactosidase, together with a slightly smaller number of genes (384) involved in protein and amino acid degradation and synthesis, were identified in the genome. Interestingly, a majority of these putative functional proteins, as well as those hypothesized to be responsible for plant growth and for the production of bioactive compounds in *Bacillus* sp. strain JS, are also present in strain D7XPN1, suggesting that these two strains may have important plant protection and degradation functions. The draft whole-genome sequence of strain D7XPN1 will assist in our understanding of the role of this particular bacterium in the food degradation process in the commercial bioreactor Baku Baku and in plant protection and growth.

### Nucleotide sequence accession numbers.

The whole-genome shotgun project of *Bacillus* species strain D7XPN1 has been deposited at DDBJ/EMBL/GenBank under the accession number JHCA00000000. The version described in this paper is version JHCA00000000.1.
